# The Clinical Burden of Inherited Neurometabolic Disorders in Adults—A Territorial Care Approach

**DOI:** 10.3390/jcm15010146

**Published:** 2025-12-24

**Authors:** Daniele Orsucci, Elena Caldarazzo Ienco, Martina Giuntini, Marco Vista

**Affiliations:** Unit of Neurology, San Luca Hospital, 55100 Lucca, Italy; elena.caldarazzoienco@uslnordovest.toscana.it (E.C.I.); martina.giuntini@uslnordovest.toscana.it (M.G.); marco.vista@uslnordovest.toscana.it (M.V.)

**Keywords:** ataxia, epileptic encephalopathies, metabolic myopathies, mitochondrial diseases, movement disorders, spastic paraparesis, stroke, stroke-like episodes

## Abstract

Neurometabolic diseases encompass a diverse group of rare and often progressive genetic disorders affecting the nervous system due to abnormalities in metabolic pathways. These conditions, including mitochondrial disorders, lysosomal storage diseases, and others, can manifest in adults with a range of neurological symptoms, which will be reviewed here. Given their complexity and chronic nature, comprehensive management is crucial for improving patients’ quality of life. In this Invited Perspective, we review the neurological signs and symptoms of the most commonly encountered inherited metabolic disorders in adult neurology. Furthermore, drawing on our clinical experience, we demonstrate that an integrated local care approach is fundamental for these patients, as it enables continuous monitoring, early intervention, and coordinated multidisciplinary support.

## 1. Introduction

Inherited metabolic diseases (IMDs) are primarily caused by genetically determined enzyme deficiencies that disrupt specific metabolic pathways. These diseases can be produced in various ways: by accumulation of the toxic substrate proximal to the block; by deficiency of the product distal to the block; or by a deviation of the substrate towards an alternative pathway [1].

Neurometabolic disorders comprise a large and diverse group of individually rare genetic conditions traditionally more familiar to pediatricians and infantile neurologists. However, adult forms have increasingly been recognized, often displaying clinical features that differ markedly from those seen in childhood and potentially mimicking more common adult neurological diseases. Importantly, in contrast to many neurogenetic conditions, a substantial number of IMDs are treatable, with therapies ranging from conventional management to newer, innovative approaches. Despite this, the diagnostic process remains challenging due to the clinical heterogeneity of these disorders and the expanding array of specialized biochemical investigations required. The biochemical diagnosis of these conditions involves multiple laboratory tests assessing specific metabolites, enzymatic activities, or functional aspects of metabolic pathways. Therapeutic strategies may aim to restore enzyme function, reduce the buildup of toxic substances, or replace deficient compounds. Early identification of a treatable neurometabolic disorder can significantly alter the clinical course by avoiding unnecessary diagnostic procedures, enabling prompt therapy, and facilitating family screening [2]. Therefore, an increased clinical awareness on these conditions has been achieved in recent years.

Moreover, next-generation sequencing (NGS) technologies have revolutionized the diagnosis of genetic neurological disorders. Exome sequencing has become widely accessible, enabling the analysis of thousands of genes within just a few weeks. These methods have significantly enhanced our overall “diagnostic yield” and are considerably more cost-effective compared to traditional Sanger sequencing. In many cases, patients do not need to visit specialized centers in person, as blood samples can be sent directly to the NGS laboratory. Even if certain limitations must be acknowledged (e.g., the vast amount and complexity of data generated by NGS making interpretation challenging), today a significant number of adult (or even elderly) patients with metabolic disorders are diagnosed using NGS techniques, often circumventing conventional biochemical testing [3].

A third contributor to the increasing number of patients with IMDs under adult neurological care is the transition into adulthood of many individuals identified as positive through neonatal biochemical screening programs since the early 2000s. Italy stands out as one of the leading countries in the screening of IMDs via dried blood spot testing in newborns, facilitating early intervention and enabling these patients to reach adulthood [4]. This development has underscored the necessity of addressing challenges related to IMDs in adult populations, including the transition and transfer of care from pediatric to adult services. To this end, the “Statement of Udine” was formulated by the adult metabolic working group of the Italian Society for the Study of Inherited Metabolic Disorders and Neonatal Screening (SIMMESN) in collaboration with the European Reference Network for Hereditary Metabolic Disorders (MetabERN), providing a framework to guide future advancements in the management of IMDs in adults in Italy [4].

In this Invited Perspective, we review the peripheral (PNS) and central nervous system (CNS) symptoms of the most commonly encountered IMDs in adult neurology. Given the complex and chronic nature of these neurometabolic disorders, comprehensive management is crucial for improving patients’ quality of life. Drawing on our clinical experience, we demonstrate that an integrated local care approach is fundamental for these patients, as it enables continuous monitoring, early intervention, and coordinated multidisciplinary support.

## 2. A Classification of IMDs

The International Classification of Inherited Metabolic Disorders (ICIMD) encompasses 1450 conditions [5]. The classification framework takes into account multiple criteria, including pathway involvement and underlying pathomechanisms. Its hierarchical, group-based structure is designed to enhance the understanding of relationships among individual disorders that may share functional, clinical, or diagnostic features. The ICIMD seeks to include any primary genetic condition in which disruption of a biochemical pathway plays a central role in defining specific biochemical, clinical, and/or pathophysiological features [5].

The ICIMD main disease groups are:

1. Disorders of amino acid metabolism
2. Disorders of peptide and amine metabolism
3. Disorders of carbohydrate metabolism
4. Disorders of fatty acid and ketone body metabolism
5. Disorders of energy substrate metabolism
6. Mitochondrial DNA (mtDNA)-related disorders
7. Nuclear-encoded disorders of oxidative phosphorylation
8. Disorders of mitochondrial cofactor biosynthesis
9. Disorders of mtDNA maintenance and replication
10. Disorders of mitochondrial gene expression
11. Other disorders of mitochondrial function
12. Disorders of metabolite repair/proofreading
13. Miscellaneous disorders of intermediary metabolism
14. Disorders of lipid metabolism
15. Disorders of lipoprotein metabolism
16. Disorders of nucleobase, nucleotide and nucleic acid metabolism
17. Disorders of tetrapyrrole metabolism
18. Congenital disorders of glycosylation
19. Disorders of organelle biogenesis, dynamics and interactions
20. Disorders of complex molecule degradation
21. Disorders of vitamin and cofactor metabolism
22. Disorders of trace elements and metals
23. Neurotransmitter disorders
24. Endocrine metabolic disorders

The first 13 categories comprise the diseases of intermediary metabolism [5], which are those disorders that involve pathways mediating the breakdown of low-molecular weight nutrient compounds belonging to one of the three major energy substrates (proteins, carbohydrates, and lipids) or convert them into substrates for the biosynthesis of complex molecules. Intermediary metabolism in the definition used in the ICIMD classification includes energy metabolism based on mitochondrial oxidative phosphorylation (categories 6–11); category 5 groups conditions that impair the availability of substrates for adenosine triphosphate (ATP) synthesis via oxidative phosphorylation.

It is not possible to discuss each individual neurometabolic disorder in detail here. Instead, the following paragraphs will summarize some of the most characteristic neurological symptoms observed in adult patients with metabolic diseases, proceeding from the PNS to the CNS.

The majority of IMDs (excluding most mitochondrial diseases, discussed in detail elsewhere) [6] exhibit an autosomal recessive inheritance pattern. Unless explicitly stated otherwise, this mode of genetic transmission is presumed when referring to individual disorders throughout the text. Table 1, Table 2, Table 3 and Table 4 provide a brief summary of the following four paragraphs and include available epidemiological data, which remain largely incomplete for most IMDs.

## 3. Myopathic Features

### 3.1. Exercise Intolerance, Muscle Pain and Rhabdomyolysis

Fatigue and exercise intolerance accompanied by muscle pain are common clinical features of IMDs. However, these complaints remain challenging to quantify objectively. This may partly explain why these manifestations are among the least studied aspects of metabolic derangements. For instance, these complaints are characteristic of mitochondrial disorders, particularly those associated with the mtDNA mutation m.3243A>G (Mitochondrial tRNA-Leu 1 deficiency), with the symptom reported in at least one-third of affected individuals [6].

Another group of disorders characterized by exercise intolerance with muscle contractures are glycogen storage diseases (GSDs), classified in ICIMD group 3. For instance, GSD5 or McArdle disease (Muscle glycogen phosphorylase deficiency) is considered to cause a ‘pure’ muscle phenotype. Exercise intolerance and episodic rhabdomyolysis are the most common symptoms, occurring in over 75% of cases [7]. The relief of myalgia and fatigue after a few minutes of rest, commonly observed in many patients, is known as the “second wind phenomenon”. This phenomenon is attributed to a metabolic shift from the impaired utilization of muscle glycogen to increased fatty acid oxidation, which becomes the primary energy source during continued exercise [8]. This compensatory mechanism enables patients with McArdle disease to resume and sustain physical activity despite the metabolic block.

Rhabdomyolysis is the clinical syndrome characterized by acute muscle necrosis with weakness and painful swelling of affected muscles, darkening of the urine due to myoglobinuria and potential kidney damage, highly elevated serum creatine kinase (CK) levels on the order of tens of thousands of IU [8]. Therapy is based on potassium-free fluid replacement, diuretics, and, in very severe cases, renal replacement therapy.

This potentially catastrophic complication (frequently triggered by anaerobic overexertion or fasting) is also a typical feature of a long-chain fatty-acid oxidation disorder (carnitine palmitoyltransferase CPT2 deficiency), under the disorders of fatty acid and ketone body metabolism (ICIMD group 4).

Other metabolic myopathies presenting with exercise intolerance and, potentially, episodic rhabdomyolysis include very long-chain acyl-CoA dehydrogenase deficiency, primary carnitine deficiency, and others [8].

### 3.2. Fixed Myopathy

“Fixed myopathy” (characterized by persistent muscle weakness and structural abnormalities, which can be confirmed by muscle biopsy, muscle MRI, myopathic EMG findings, and/or elevated CK levels) is more typical of other genetic conditions (e.g., dystrophinopaties) but can be also a feature of several IMDs.

For instance, GSD2 (Pompe disease or Alpha-glucosidase deficiency), a potentially treatable condition, typically presents with proximal muscle weakness, at least in the intermediate and late stages of the disease.

In advanced stages, in most metabolic myopathies there might be persistent and progressive muscle weakness [8].

A particular subtype of fixed myopathy is ocular myopathy, characterized by eyelid ptosis and, eventually, ophthalmoparesis. This presentation is typical of several mitochondrial disorders. Ocular myopathy is also referred to as progressive external ophthalmoplegia (PEO) when it represents the main clinical feature. PEO is the most frequent mitochondrial phenotype, occurring in approximately half of patients with a confirmed molecular diagnosis. It is more strictly associated with sporadic single large-scale mtDNA deletions or autosomal dominant or recessive *POLG* mutations. Although there is still a strong need for more homogeneous patient categorization, the term pure PEO has been suggested to identify patients with isolated ocular myopathy, whereas PEO plus refers to those with ocular myopathy and additional neuromuscular features. Patients with ocular myopathy and evidence of structural CNS dysfunction should not be classified as having PEO, but rather as presenting with a more complex mitochondrial encephalomyopathy [9].

### 3.3. Metabolic Cardiomyopathies

IMDs often present with multisystemic involvement, and cardiac manifestations are among the most frequent. Cardiomyopathies, particularly hypertrophic cardiomyopathy, are common and can be associated with various IMDs, including hereditary haemochromatosis, Fabry disease, Pompe disease, Danon disease, Friedreich’s ataxia, mitochondrial disorders, very long-chain acyl-CoA dehydrogenase deficiency, and carnitine palmitoyltransferase II deficiency. A comprehensive overview of cardiac involvement in IMDs is available elsewhere [1]. IMDs should always be considered in the differential diagnosis of cardiomyopathies of unknown origin.

In mitochondrial diseases, approximately 30% of adult patients present with ECG and/or echocardiographic abnormalities. Cardiac involvement is associated with a poorer prognosis. Early detection of cardiomyopathy is a critical component of both pediatric and adult management when a mitochondrial disease is known or suspected. A prediction model was developed through a retrospective study of 600 adult mitochondrial patients from a multicenter European registry. Over a median follow-up of 6.7 years, 5% of patients reached the heart failure endpoint, and another 5% experienced arrhythmic events. Predictors of heart failure included the m.3243A>G variant, conduction abnormalities, left ventricular hypertrophy, reduced ejection fraction (<50%), and premature ventricular contractions. Independent predictors of arrhythmic events were single large-scale mtDNA deletions, conduction abnormalities, and ejection fraction <50%. Overall, cardiac outcomes were significantly worse in patients harboring the m.3243A>G variant or single large-scale mtDNA deletions compared with those carrying other mitochondrial variants [10].

## 4. Peripheral Nervous System Complaints

### 4.1. Peripheral Neuropathy

Mitochondrial disorders involving the PNS, with motor and/or sensory complaints, are well known. The prevalence of clinically apparent peripheral neuropathy may exceed 10% in this group of diseases. For instance, mutations in the nuclear gene *POLG* (Mitochondrial DNA polymerase gamma catalytic subunit deficiency) can cause a painful, predominantly sensory axonal or mixed polyneuropathy. The only mtDNA mutation associated with neuropathy is the m.8993T>G (or T>C) “NARP” (neuropathy, ataxia, and retinitis pigmentosa syndrome) substitution (Mitochondrial ATP synthase F0 subunit 6 deficiency), which causes a predominantly sensory axonal polyneuropathy and is transmitted via maternal inheritance [6].

One of the most common inherited axonal neuropathies, Charcot-Marie-Tooth disease type 2A (CMT2A), is caused by dominant mutations in *MFN2* (Mitofusin 2 deficiency), classified by the ICIMD under group 19 (Disorders of organelle biogenesis, dynamics, and interactions). MFN2 is a GTPase anchored to the outer mitochondrial membrane, involved in maintaining the balance between mitochondrial fusion and fission, as well as other mitochondrial processes. CMT2A is clinically characterized by severe neuropathy, primarily affecting motor function, and in some cases, accompanied by significant loss of proprioception. This leads to progressive muscle weakness and atrophy in the legs and arms, with onset typically occurring during childhood and resulting in considerable disability [11].

Other, rarer causes of neuropathy related to mitochondrial dysfunction are included among the disorders of mitochondrial aminoacyl-tRNA synthetases, categorized by the ICIMD under group 10 (Disorders of mitochondrial gene expression).

### 4.2. Small Fiber and Enteric Neuropathies

The autonomic nervous system controls involuntary body functions. Its major divisions are the sympathetic, parasympathetic, and enteric nervous systems. Autonomic fibers are predominantly small unmyelinated fibers, involved in regulating visceral and vascular functions. In addition to autonomic fibers, “small fibers” mediate pain, temperature sensation, itch, and various autonomic functions including sweating and gastrointestinal motility.

Hereditary sensory and autonomic neuropathies (HSAN) comprise a group of inherited neuropathies characterized by both sensory and autonomic involvement. Some forms are metabolic in nature. For example, HSAN1A and HSAN1C are caused by autosomal dominant deficiencies in serine palmitoyltransferase subunits 1 and 2, respectively, and are classified among lipid metabolism disorders in ICIMD group 14. Clinical onset is typically marked by impaired pain and temperature sensation in the distal extremities, resulting from small fiber dysfunction. This sensory loss often predisposes to recurrent infections, which may evolve into osteomyelitis and eventually necessitate amputations [12]. Autonomic and motor involvement is variable.

HSAN3, or familial dysautonomia, is caused by recessive mutations in the *ELP1* gene (ICIMD group 16: disorders of nucleobase, nucleotide, and nucleic acid metabolism). The condition presents with reduced pain and temperature sensation, recurrent hyperadrenergic autonomic crises (vomiting, hypertension, tachycardia), afferent baroreflex failure with severe blood pressure variability, neurogenic orthostatic hypotension, progressive sensory ataxia, and visual loss. Morbidity and mortality are often linked to sleep-disordered breathing, chronic lung disease, and sudden death during sleep. Gastrointestinal symptoms (dysphagia, reflux, nausea) are common, and patients typically have reduced weight, height, and body mass index compared to the general population [13].

Moreover, gut dysmotility is a feature of several mitochondrial encephalomyopathies, for instance those caused by mutations in *TYMP* and *POLG*. Mitochondrial neurogastrointestinal encephalomyopathy (MNGIE) is a very rare, devastating autosomal recessive mitochondrial disease caused by thymidine phosphorylase deficiency due to *TYMP* mutations, which usually leads to death in early adulthood. One of the major clinical features is progressive gastrointestinal dysmotility due to enteric neuropathy, with diarrhea, abdominal pain, vomiting, pseudo-obstruction, and cachexia. Peripheral neuropathy, leukoencephalopathy, and ocular myopathy are also commonly observed [14]. *TYMP*-associated MNGIE is one of the few mitochondrial diseases potentially responsive to treatment, aimed at counterbalancing the toxic accumulation of thymidine nucleosides.

A very similar (“MNGIE-like”) phenotype has recently been described in patients with mutations in mitochondrial ligase III (*LIG3*), which is crucial for mtDNA repair and replication, and thus for mitochondrial function. A decreased number of enteric neurons was reported in these patients [15]. Both conditions affect mtDNA maintenance, leading to mtDNA depletion.

### 4.3. Lower Motor Neuron Involvement

Kennedy’s disease, also known as spinal and bulbar muscular atrophy (SBMA), is an X-linked neuromuscular disorder classified under group 24, “Endocrine metabolic disorders” (ICIMD). It is clinically characterized by progressive weakness, atrophy, and fasciculations of the limb and bulbar muscles, resulting from lower motor neuron degeneration. The condition is caused by an abnormal expansion of CAG trinucleotide repeats in the ubiquitously expressed androgen receptor (*AR*) gene. In addition to motor symptoms, SBMA often presents with multisystem involvement, including myopathy, sensory axonal neuropathy, gynecomastia, impotence, and testicular atrophy [16].

## 5. Impairment of Special Senses

### 5.1. Retinopathy

The involvement of non-neural ocular structures in IMDs (e.g., corneal pathology in Fabry disease, Gaucher disease, and mucopolysaccharidoses; early bilateral cataracts in Wilson disease, cerebrotendinous xanthomatosis, and galactosemia; dislocation of the lens in classical homocystinuria, etc.) will not be discussed here, as it has been excellently reviewed elsewhere [17].

The retina is a highly metabolically active tissue; thus, defects in energy metabolism can have a dramatically detrimental effect. Retinal degeneration, especially retinal dystrophy caused by monogenic inherited disorders, is often characterized by early-onset photoreceptor cell death, leading to progressive loss of vision. Groups of IMDs that often present with retinal degeneration include neuronal ceroid lipofuscinoses, iminoglycinuria, congenital disorders of glycosylation, complex lipid disorders, as well as disorders of copper metabolism and cobalamin C deficiency [17].

Retinopathy is a characteristic feature of a few mitochondrial diseases. In particular, pigmentary retinopathy is one of the defining features of NARP (neuropathy, ataxia, retinitis pigmentosa) syndrome, caused by *MT-ATP6* mutations in mtDNA, and of Kearns-Sayre syndrome, which affects carriers of sporadic large-scale mtDNA deletions [6].

Some IMDs present with isolated ocular manifestations, often due to deficiencies in enzymes involved in vitamin A metabolism and, consequently, in the visual cycle (e.g., *RPE65*, *LRAT*, *RDH12*, etc.). Pathogenic variants in these genes cause Leber congenital amaurosis, a congenital and severe form of retinal disease. Another example of an IMD with a predominant ocular phenotype is ornithine aminotransferase deficiency, which primarily affects the retina and, secondarily, the choroid, leading to “gyrate atrophy” [17].

### 5.2. Optic Neuropathy

The inherited optic neuropathies most frequently encountered in adult neurological practice belong to the group of IMDs, specifically mitochondrial diseases.

Leber hereditary optic neuropathy (LHON) is a maternally transmitted disorder characterized by subacute, painless, bilateral central vision loss that primarily affects young males. The three primary LHON-associated pathogenic mtDNA mutations are the homoplasmic m.3460G>A, m.11778G>A, and m.14484T>C. Gene-environment interactions play a significant role. A large, multicenter study involving 196 affected and 206 unaffected carriers from 125 LHON pedigrees retrospectively examined exposure to smoking, alcohol, and other environmental factors [18]. This study identified a strong and consistent association between visual loss and smoking, independent of gender, leading to a high penetrance (>90%) in male smokers. There was also a trend toward increased visual failure with alcohol consumption, though only with heavy intake. Based on these findings, asymptomatic carriers of a LHON mtDNA mutation should be strongly advised to avoid smoking and to moderate their alcohol intake [6]. A registry study of LHON involving 620 affected individuals and 4948 asymptomatic carriers showed that the minimum prevalence of vision loss due to LHON in Australia in 2020 was approximately 1.5 per 100,000 individuals. The overall risk of vision loss among those with a LHON mutation was lower than previously reported (18% for males and 5% for females) [19]. Accurate knowledge of this risk is essential for the genetic counseling of individuals with LHON mutations [6].

Mutations in the nuclear-encoded *OPA1* gene are the most common cause of autosomal dominant optic atrophy (ADOA), a major cause of inherited visual failure. ADOA is included in the ICIMD Group 19 (Disorders of organelle biogenesis, dynamics, and interactions). A large multicenter study involving >100 patients introduced the concept of “ADOA plus” [20]. This study demonstrated that extra-ocular neurological complications affected up to 20% of all mutation carriers. The most prominent additional manifestation was bilateral sensorineural deafness, typically beginning in late childhood or early adulthood. This was followed by combinations of ataxia, myopathy, peripheral neuropathy, and external ophthalmoplegia starting from the third decade of life. Rarer clinical presentations included spastic paraparesis and multiple sclerosis-like syndromes. Studies on skeletal muscle biopsies revealed cytochrome c oxidase (COX)-deficient fibers and multiple mtDNA deletions in most patients. Individuals with the “dominant optic atrophy plus” phenotype also showed significantly worse visual outcomes compared to those with isolated optic atrophy [20].

### 5.3. Sensorineural Hearing Loss

Sensorineural hearing loss is a relatively common feature in patients with IMDs, with prevalence ranging from isolated case reports (e.g., Pompe disease) to nearly universal involvement in certain conditions (e.g., β-mannosidosis or thiamine-responsive megaloblastic anemia). The damage typically affects the inner ear, as in propionic acidemia, but may also involve retrocochlear pathways, such as auditory nerve demyelination seen in biotinidase deficiency. Tinnitus may also be present [21].

Moreover, sensorineural hearing loss is a common feature in several mitochondrial diseases. A retrospective analysis was conducted on DNA samples from the U.S. National Hereditary Deafness DNA Repository to identify mitochondrial mutations [22]. Out of the total sample, 86 patients (3.5%) had mitochondrial mutations. Among these, 24% carried the m.7445A>G mutation, 21% had m.1555A>G, another 21% had m.961T>G, and 34% presented with a mtDNA deletion. Most patients showed bilateral severe hearing loss. Females accounted for 62% of the cases, and 77% had a family history of hearing loss. Since some mtDNA mutations increase sensitivity to aminoglycosides, accurate diagnosis is essential to prevent potential harm to matrilineal relatives [22].

## 6. Central Nervous System Pathology

### 6.1. Hyperkinetic Movement Disorders

Hyperkinetic movement disorders represent a heterogeneous group of conditions characterized by excessive, involuntary motor activity, each with distinct clinical presentations and underlying pathophysiological mechanisms.

Hyperekplexia (HPX), also known as Startle disease, is a rare neurometabolic disorder clinically characterized by generalized muscle stiffness and an exaggerated startle response beginning in infancy. This response is typically triggered by sudden and unexpected auditory, tactile, visual, or even emotional stimuli. The pathological startle reaction may persist throughout life and can lead to significant social impairment and risk of traumatic injury. Despite its potential severity, HPX generally has a favorable prognosis, largely due to the effectiveness of clonazepam in controlling startle episodes [23]. The therapeutic benefit of benzodiazepines is thought to result from their ability to enhance GABA A receptor–mediated chloride currents, which may partially compensate for the impaired function of glycine-gated chloride channels seen in HPX. Among the genes implicated in HPX, *GLRA1*, which encodes the α1 subunit of the glycine receptor, is the most frequently mutated, accounting for approximately 80% of cases. Both autosomal dominant and autosomal recessive inheritance patterns have been reported. HPX caused by *GLRA1* mutations (Glycine receptor subunit alpha 1 deficiency) is classified under Group 23 (Neurotransmitter disorders) in the ICIMD nosology. Notably, clinical expressivity is highly variable, even among individuals carrying the same pathogenic variant [23]. Late-onset and/or atypical cases have been reported [24].

Myoclonus refers to a sequence of repeated, brief, shock-like jerks that are typically non-rhythmic and result from sudden, involuntary contraction or relaxation of one or more muscles. In some cases, it occurs as part of a group of symptomatic generalized epilepsies collectively known as progressive myoclonus epilepsies, characterized by a disabling clinical course and poor prognosis. The less rare form (especially in adults) is represented by “myoclonus epilepsy with ragged-red fibers” (MERRF) syndrome, a maternally inherited, mtDNA associated mitochondrial disorder (Mitochondrial tRNA-Lys deficiency, ICIMD). However, in mitochondrial diseases, myoclonus is often more closely associated with ataxia than with generalized seizures, suggesting that mitochondrial myoclonus may represent a subcortical movement disorder rather than a classical epileptic encephalopathy [6]. Other forms of progressive myoclonic epilepsies, including type 2a (Laforin deficiency), type 2b (Malin deficiency), type 3 (CLN14 disease), type 4 (Glucocerebrosidase receptor deficiency), and type 8 (Ceramide synthase 1 deficiency), are classified within various subgroups of IMDs.

Dystonia is a movement disorder characterized by sustained or intermittent muscle contractions, resulting in abnormal and often repetitive movements or postures. Brief involuntary movements, such as chorea and athetosis, are commonly associated with dystonia. These conditions can be a feature of various IMDs, including autosomal dominant or recessive GTP cyclohydrolase-1 deficiency (Dopa-responsive dystonia), glutaryl-CoA dehydrogenase deficiency (Glutaric acidemia type 1), pantothenate kinase 2 deficiency (neurodegeneration with brain iron accumulation type 1), copper-transporting ATPase subunit beta deficiency (Wilson disease), hypoxanthine-guanine phosphoribosyl transferase deficiency (Lesch-Nyhan disease), methylmalonic aciduria, propionic acidemia, and severe pediatric encephalomyopathies (e.g., Leigh syndrome), among others [25]. Chorea-acanthocytosis is also considered an IMD, included within the subgroup of disorders of organelle interplay (ICIMD); this condition typically presents in early adulthood with involuntary choreiform movements and red blood cell acanthocytosis [26].

Furthermore, tremor, defined as an involuntary, rhythmic, oscillatory movement of a body part, can be a feature of IMDs (e.g., mitochondrial), although isolated tremor is rare. Most commonly, it is not a truly hyperkinetic disorder but rather a “hypostenic” myopathic and/or neuropathic tremor [27]. A postural or kinetic tremor may be the first sign of cerebellar ataxia, whereas a resting tremor can represent the onset of parkinsonism (see next paragraphs).

### 6.2. Cerebellar Ataxia

Ataxia is characterized by incoordination or imbalance (e.g., truncal ataxia, gait ataxia) of a limb while executing a task (dysmetria), and is usually caused by dysfunction of the cerebellum or its connections. However, sensory PNS involvement may also cause ataxia. Neurological examination allows a correct distinction between the two conditions. Furthermore, cerebellar ataxia is frequently associated with other signs of cerebellar dysfunction, including abnormal eye movements, dysmetria, kinetic tremor, dysarthria, and/or dysphagia [9].

Ataxia is one of the clinical symptoms of several well-defined mitochondrial syndromes, including Kearns–Sayre syndrome; maternally inherited Leigh syndrome; MERRF; and NARP. It may also be present in undefined mitochondrial encephalopathies. Even though cerebellar ataxia can be the presenting symptom of one of these mtDNA-associated disorders, discussed in detail elsewhere [9], isolated ataxia may also result from autosomal recessive defects in intergenomic signaling. These include, in particular, *POLG*-related diseases (i.e., ataxia-myopathy syndrome; “mitochondrial recessive ataxia syndrome,” or MIRAS; ataxia with ocular myopathy and, more rarely, with psychiatric comorbidities or epilepsy) and certain *C10orf2* (“Twinkle”) mutations, which can cause infantile-onset spinocerebellar ataxia (IOSCA), a severe neurodegenerative disorder characterized by progressive atrophy of the brainstem, cerebellum, and spinal cord, and by sensory axonal neuropathy. Ataxia can also be the first symptom in rare patients with ADOA due to *OPA1* gene mutations [9].

Furthermore, isolated cerebellar ataxia is one of the typical features of autosomal recessive coenzyme Q10 deficiencies. Coenzyme Q10 biosynthetic genes (e.g., *COQ8A*) are the primary candidates in patients with progressive cerebellar ataxia and cerebellar atrophy on imaging studies [3]. Coenzyme Q10 deficiency is a treatable condition; therefore, timely diagnosis is essential.

Cerebellar involvement is typical of many IMDs other than mitochondrial ones, and it can be part of the clinical picture in about half of them. The forms that can be encountered in the adult setting, although rarely, include other potentially treatable conditions, such as ataxia with vitamin E deficiency and Niemann-Pick disease type C [25].

Other forms of ataxia considered to be of metabolic origin include spastic ataxia of Charlevoix-Saguenay (Sacsin deficiency), episodic ataxia type 6 (Glutamate aspartate transporter deficiency), ataxia-oculomotor apraxia type 3, and various forms of autosomal recessive cerebellar ataxias.

Friedreich ataxia is also frequently regarded as a metabolic disorder due to its association with mitochondrial dysfunction, oxidative stress, and impaired iron metabolism. It is an autosomal recessive condition caused by mutations in the *FXN* gene, most commonly a biallelic expansion of GAA trinucleotide repeats, and affects approximately 1–2 per 100,000 individuals. The disease typically presents between 7 and 15 years of age, although onset can be variable. Clinical manifestations include progressive ataxia, sensory loss, scoliosis, hypertrophic cardiomyopathy, optic atrophy, and diabetes mellitus. The length of the shorter GAA repeat correlates with frataxin expression and disease severity, but this alone does not account for all the observed variability in age at onset and disease progression [28].

### 6.3. Parkinsonism

Parkinsonism (a hypokinetic movement disorder) is a significant finding in adult-onset mitochondrial diseases. Nearly 70% of patients with mitochondrial parkinsonism responded to dopaminergic therapy, primarily L-dopa, in a manner similar to patients with idiopathic Parkinson’s disease [27].

The cardinal features of Parkinson’s disease include resting tremor, rigidity, bradykinesia and postural instability. The symptoms are caused by the progressive loss of the dopamine neurons within the substantia nigra pars compacta and the associated deficiency of the neurotransmitter dopamine in the striatum. The case of a Parkinson’s disease patient with some signs or symptoms suggestive of mitochondrial disease (i.e., ptosis, myopathy, neuropathy) is a relatively common event in the adult neurological practice. Mitochondrial parkinsonisms (e.g., due to *POLG* mutations) do not have distinctive features allowing an immediate diagnosis, and a negative family history does not rule out a possible diagnosis of mitochondrial disorder [9]. In these cases, molecular studies are mandatory.

Furthermore, recessive early-onset Parkinson’s disease type 2 (Parkin-PRKN-deficiency) and type 6 (PINK1 deficiency) are classified in group 11 of the ICIMD nosology (“Other disorders of mitochondrial function”), while early-onset Parkinson’s disease type 20 (Synaptojanin 1 deficiency) is included in group 14 (“Disorders of lipid metabolism”). The autosomal recessive Parkinson’s disease type 23 (VPS13C deficiency) and the autosomal dominant type 22 (CHCHD2 deficiency) are listed under group 19 (“Disorders of organelle biogenesis, dynamics and interactions”), whereas type 9 (ATP13A2 deficiency, also recessive) falls under group 20 (“Disorders of complex molecule degradation”).

Interestingly, different variants in the *GBA1* gene (associated with Gaucher disease) can act as genetic risk factors for parkinsonism or even cause a monogenic form of Parkinson disease with reduced penetrance (PARK-GBA1).

### 6.4. Spastic Paraparesis (or Tetraparesis)

Spasticity is an increase in muscle tone that reflects pyramidal tract dysfunction and is often accompanied by other pyramidal signs, including hyperreflexia, clonus, and the Babinski sign. Upper motor neurons are particularly vulnerable to metabolic disruption due to their high energy demands, making spasticity a common feature of many neurometabolic disorders. However, spasticity rarely occurs as an isolated manifestation among IMDs [25], and moderate-to-severe intellectual disability may also be present. Among the forms presenting with isolated spastic paraparesis, type 7 (paraplegin deficiency) is classified as an IMD according to the ICIMD classification, along with other, rarer forms of spastic paraparesis (types 5a, 9, 11, 13, 15, 17 and others).

Furthermore, spasticity is a core clinical feature of leukodystrophies. X-linked adrenoleukodystrophy (or adrenomyeloneuropathy) is the most common peroxisomal disorder encountered in clinical practice and is likely the metabolic leukodystrophy most frequently diagnosed in adults. Psychiatric manifestations, optic atrophy, and hearing impairment are commonly associated features of this disease. Addison’s disease may also be present, and the clinical course can be accelerated by trauma or intercurrent illnesses. Adult-onset presentations of other potentially similar neurometabolic conditions, namely metachromatic leukodystrophy and Krabbe disease, are rarer [29].

### 6.5. Stroke

Stroke is a well-recognized neurological complication of Fabry disease, an X-linked lysosomal storage disorder caused by pathogenic variants in the *GLA* gene, leading to deficiency of the alpha-galactosidase A enzyme (α-Gal), which is involved in glycosphingolipid metabolism. As a result, globotriaosylceramide (Gb3), globotriaosylsphingosine (LysoGb3), and other lesser-known catabolites progressively accumulate in lysosomes and other subcellular compartments of various cell types, including neurons, endothelial and smooth muscle cells, cardiomyocytes, and specialized cells of the cardiac conduction system. Fabry disease is characterized by marked phenotypic heterogeneity. The degree of residual α-Gal activity influences symptom onset and clinical presentation. In males, absent or near-absent enzyme activity results in the classic phenotype, characterized by early-onset acroparesthesias, angiokeratomas, hypohidrosis, and progressive renal, cardiac, and cerebrovascular involvement. Reduced enzyme activity (up to 20% of normal) is associated with the late-onset form. In females, clinical manifestations are highly variable due to random X-chromosome inactivation. Multiple mechanisms have been implicated in the pathogenesis of ischemic stroke in Fabry patients, including dolichoectasia of cerebral arteries, small vessel disease, cardiogenic embolism, autonomic dysfunction, and alterations in blood composition. However, *GLA* variants are found in fewer than 1% of patients with ischemic stroke aged 18–60 years. Predictors of *GLA* variants may include mild stroke severity, recurrence, history of transient ischemic attack, acroparesthesias, hearing loss, and small artery occlusion [30].

### 6.6. Stroke-like Episodes

A stroke-like event is an evolving subacute neurological syndrome associated with seizure activity and focal metabolic brain dysfunction. While the sudden onset of focal neurological deficits (e.g., hemiparesis) evolving rapidly within minutes typically points to a vascular stroke, the subacute development (over hours) of complex visual symptoms and/or altered mental status may indicate an ongoing metabolic stroke-like episode. Headache and visual disturbance are often experienced during the prodrome.

Stroke-like episodes in individuals with mitochondrial disease typically present with prodromal symptoms such as headache, nausea and vomiting, altered consciousness, focal-onset seizures, or psychiatric manifestations, followed by the development of a focal neurological deficit. Epilepsia partialis continua and, rarely, generalized status epilepticus might also occur during the stroke-like episode [31].

Commonly available investigations such as brain MRI, EEG, and laboratory tests (including serum lactate) can assist in establishing the correct diagnosis. The most frequent cause of stroke-like episodes is a mitochondrial disease (MELAS: mitochondrial encephalopathy, lactic acidosis, and stroke-like episodes syndrome). MELAS is commonly due to the m.3243A>G mtDNA mutation, followed by recessive pathogenic variants in the nuclear *POLG* gene, and other rarer mtDNA mutations [6].

### 6.7. Epileptic Encephalopathies

Seizures are a common manifestation of IMDs, although they appear to be less frequent in adults than in pediatric patients. A detailed review of early infantile-onset epileptic encephalopathies is beyond the scope of this review and can be found elsewhere [32]. Most of these patients also have moderate-to-severe intellectual disability and other CNS symptoms (e.g., spastic tetraparesis).

Studies investigating epilepsy in patients with mitochondrial disorders are limited. In the database of the Italian network, 10% of patients were reported to have epilepsy [33].

Some cases of neuronal ceroid lipofuscinoses (NCLs) (ICIMD group 20, Disorders of complex molecule degradation) may present with seizures in adulthood [32,34]. NCLs (also known as “Batten disease”) are a group of lysosomal storage disorders characterized by the excessive accumulation of lipofuscin in multiple tissues, with a devastating impact on the CNS. Clinically, NCLs typically manifest in infancy with vision impairment, motor and cognitive dysfunction, epilepsy, and premature death [35], although some patients may survive into adulthood. Less commonly, NCLs may present in early adulthood, with symptoms including early-onset dementia, myoclonic seizures, movement abnormalities (such as ataxia and pyramidal or extrapyramidal signs), psychiatric disturbances, and visual loss [35].

Another metabolic encephalopathy that may manifest with seizures from infancy to adulthood [32] is Succinic Semialdehyde Dehydrogenase Deficiency (SSADHD), a neurotransmitter disorder classified under ICIMD group 23. The accumulation of GABA and GABA-related metabolites leads to a broad spectrum of CNS disturbances, including cognitive impairment, movement disorders, epilepsy, and sleep disturbances [34].

## 7. Care Standards (and Our Experience)

Beyond the pharmacological management of the various clinical manifestations (and, in a few cases, the specific metabolic defect), standards of care for IMDs include the use of digital hearing aids for sensorineural hearing loss, pacemakers and/or implantable defibrillators for cardiac arrhythmias, more rarely deep brain stimulation for refractory movement disorders, etc.

Potential triggers of metabolic decompensation should be promptly identified and managed, including diabetic ketoacidosis, alcohol or other toxic exposures, diarrhea, prolonged fasting, and infections. In most cases, patients with IMDs should receive age-appropriate vaccinations.

Comprehensive care also encompasses enteral nutrition and/or assisted ventilation for patients with advanced disease. Swallowing difficulties are common and may arise from multiple mechanisms, including oropharyngeal muscle weakness due to neuromuscular impairment, incoordination related to cerebellar ataxia or involuntary movements, or bradykinesia and cognitive impairment associated with parkinsonism and psychomotor regression. Severe neuromuscular weakness with diaphragmatic involvement, requiring ventilatory support, is less frequent but may occur.

A multidisciplinary team, typically including, among others, a pulmonologist, phoniatrist, and cardiologist, coordinated by the neurologist acting as case manager, is essential for the early recognition and management of these complications. A comprehensive rehabilitative program is strongly recommended to preserve quality of life and should extend beyond the mere prescription of assistive devices. Physical, occupational, and speech therapy, along with attention to general care including optimization of nutritional needs and swallowing issues, improve patient outcomes and quality of life [29].

A dedicated focus on the transition from pediatric to adult care is imperative. Health care professionals, including pediatricians, child neuropsychiatrists, and neurologists, who are responsible for the continuity of neurological management in these vulnerable adolescents and young adults, should receive appropriate training and engage in close interdisciplinary collaboration to ensure a seamless transition of care [36].

Psychologists and social workers are also frequently involved in the long-term follow-up of these patients. In advanced disease stages, palliative care may be indicated. For the most severely affected individuals, neurological assessments and most other medical consultations should be conducted at home.

In our local experience, patients with more advanced disease (i.e., a modified Rankin Scale score of 5) [37] are followed up at home (Table 5). In this context, it is crucial to have a local team trained and competent in managing rare neurological disorders in adults, including those of metabolic origin, as relying exclusively on distant university centers would not allow for optimal patient care.

## 8. Conclusions

Neurometabolic diseases in adults are individually rare, numerous, heterogeneous and frequently show complex clinical presentations [2].

The classification and management of adult neurometabolic disorders are often extrapolated from pediatric literature, where diagnostic and therapeutic standards are more established. Although there is a clinical continuum from childhood to adulthood, with milder phenotypes often emerging later in life, certain inborn errors of metabolism occur predominantly in adults. Several acquired conditions in this age group, including infections, immune-mediated diseases, and toxin exposures, can mimic the clinico-radiological presentation of IMDs (e.g., leukoencephalopathies) [29]. Therefore, ruling out acquired causes remains a cornerstone of the initial diagnostic evaluation.

A detailed three-generation family history, careful documentation of the chronology of neurological symptoms and signs, identification of relevant systemic associations, and pattern recognition on neuroimaging frequently provide key insights into the underlying etiology. Early diagnosis is critical, as several IMDs are potentially treatable when recognized in the initial stages of the disease course [29].

Molecular confirmation is also of great diagnostic value, and advances in high-throughput sequencing technologies have significantly reshaped the diagnostic workflow for IMDs, increasingly favoring a genetics-first approach. Whole-exome or even whole-genome testing should be offered early in the diagnostic odyssey of these patients. In most cases, referral to a distant center is unnecessary, as the sample can be collected locally and sent to the reference laboratory.

We believe that an integrated local care approach is essential for these patients, as it may enable early diagnosis, continuous monitoring, timely intervention, and coordinated multidisciplinary support, including home-based care when needed.

## Figures and Tables

**Table 1:** **Myopathic features**. Based on the information discussed in the text and on https://www.orpha.net (accessed on 17 December 2025) for epidemiological data. AR, autosomal recessive; MI, maternal inheritance; mtDNA, mitochondrial genome; N.A. specific data not available on Orphanet; PEO, progressive external ophthalmoplegia.

Sign/Symptom	Examples of Diseases	Gene(s)	Associated Features	Epidemiology (Prevalence)
Exercise intolerance	Mitochondrial disorders	*MTTL1* (MI) and others	Extremely polymorphic	N.A.
Exercise intolerance and episodic rhabdomyolysis	McArdle disease	*PYGM* (AR)	Second wind phenomenon	1/100,000 to 1/170,000
Episodic rhabdomyolysis	CPT2 deficiency	*CPT2* (AR)	-	1–9/100,000
Fixed myopathy	Pompe disease	*GAA* (AR)	Proximal muscle weakness	N.A.
Ocular myopathy (PEO)	Mitochondrial disorders	mtDNA deletions (sporadic), others	Extremely polymorphic	N.A.
Cardiomyopathy	Mitochondrial disorders	*MTTL1* (MI) and others	Extremely polymorphic	N.A.

**Table 2:** **Peripheral nervous system complaints**. Based on the information discussed in the text and on https://www.orpha.net (accessed on 17 December 2025) for epidemiological data. AD, autosomal dominant; AR, autosomal recessive; CMT, Charcot-Marie-Tooth; HSAN, Hereditary sensory and autonomic neuropathies; MNGIE, Mitochondrial neurogastrointestinal encephalomyopathy; N.A. specific data not available on Orphanet.

Sign/Symptom	Examples of Diseases	Gene(s)	Associated Features	Epidemiology (Prevalence)
Peripheral neuropathy	Mitochondrial disorders	*POLG* (AD or AR) and others	Extremely polymorphic	N.A.
Peripheral neuropathy	CMT2A	*MFN2* (AD)	Axonal neuropathy, proximal myopathy	N.A.
Small fiber neuropathy	HSAN1A, HSAN1C	*SPTLC1-2* (AD)	Impaired pain and temperature sensation, infections, osteomyelitis	<1/1,000,000
Small fiber neuropathy	Familial dysautonomia	*ELP1* (AR)	Autonomic crises, orthostatic hypotension, sudden death	<1/1,000,000
Gut dysmotility	MNGIE	*TYMP* (AR)	Peripheral neuropathy, leukoencephalopathy, ocular myopathy	1–9/1,000,000
Lower motor neuron disease	Kennedy’s disease	*AR* (X-linked)	Sensory neuropathy, gynecomastia	1–9/100,000

**Table 3:** **Impairment of special senses**. Based on the information discussed in the text, and on https://www.orpha.net (accessed on 17 December 2025) for epidemiological data. AD, autosomal dominant; MI, maternal inheritance; mtDNA, mitochondrial genome; N.A. specific data not available on Orphanet; NARP, neuropathy, ataxia, retinitis pigmentosa syndrome; PEO, progressive external ophthalmoplegia.

Sign/Symptom	Examples of Diseases	Gene(s)	Associated Features	Epidemiology (Prevalence)
Retinopathy	Mitochondrial disorders (e.g., NARP)	*MTATP6* (MI) and others	Extremely polymorphic	1–9/100,000 (NARP)
Optic neuropathy	Leber optic neuropathy	*MTND1,4,6* (MI)	Subacute vision loss (especially male smokers)	1–9/100,000
Optic neuropathy	AD optic atrophy (ADOA)	*OPA1* (AD)	Deafness, PEO	1–9/100,000
Sensorineural hearing loss	Mitochondrial disorders	mtDNA deletions (sporadic), others	Extremely polymorphic	N.A.

**Table 4:** **Central nervous system pathology**. Based on the information discussed in the text and on https://www.orpha.net (accessed on 17 December 2025) for epidemiological data. AD, autosomal dominant; AR, autosomal recessive; HSP, hereditary spastic paraparesis; MELAS, mitochondrial encephalopathy, lactic acidosis, and stroke-like episodes syndrome; MERRF, myoclonus epilepsy with ragged-red fibers syndrome; MI, maternal inheritance; mtDNA, mitochondrial genome; N.A. specific data not available on Orphanet; SSADHD, Succinic Semialdehyde Dehydrogenase Deficiency.

Sign/Symptom	Examples of Diseases	Gene(s)	Associated Features	Epidemiology (Prevalence)
Exaggerated startle response	Hyperekplexia	*GLRA1* (AD or AR)	Excellent response to benzodiazepines	<1/1,000,000
Myoclonus	MERRF	*MTTK* (MI)	Cerebellar ataxia, myopathy	N.A.
Chorea, dystonia	Chorea-acanthocytosis	*VPS13A* (AR)	Myopathy	N.A.
Cerebellar ataxia	Mitochondrial disorders	*POLG* (AD, AR) and others	Extremely polymorphic	N.A.
Cerebellar ataxia	Coenzyme Q10 deficiency	*COQ8A* (AR) and others	Myopathy	N.A.
Cerebellar ataxia	Friedreich ataxia	*FXN* (AR)	Sensory loss, cardiomyopathy, diabetes	1–9/100,000
Parkinsonism	Mitochondrial disorders	*POLG* (AD, AR) and others	Extremely polymorphic	N.A.
Spasticity	HSP7	*SPG7* (AR)	Cerebellar ataxia	N.A.
Spasticity	X-linked adrenoleukodystrophy	*ABCD1* (X-linked)	Psychiatric manifestations, optic atrophy, hearing impairment, Addison	1/17,000 (birth prevalence)
Stroke	Fabry disease	*GLA* (X-linked)	Acroparesthesias, hearing loss, cardiomyopathy, renal involvement	1–5/10,000
Stroke-like episodes	MELAS	*MTTL1* (MI)	Headache, nausea, altered consciousness, focal-onset seizures	N.A.
Epileptic encephalopathy	Neuronal ceroid lipofuscinoses	*CLN5* (AR) and others	Vision impairment, cognitive dysfunction, spastic tetraparesis, myoclonus	<1/1,000,000 (CLN5)
Epileptic encephalopathy	SSADHD	*ALDH5A1* (AR)	Cognitive impairment, movement disorders, sleep disturbances	1–9/1,000,000

**Table 5:** **Our experience with neurometabolic disorders**. IMD patients represented 18.9% (43/228) of the subjects with a known or newly diagnosed monogenic condition evaluated in our Rare Neurological Disease outpatient center (serving a territory with ~220.000 persons) during the last year. Individually recognizable data that are not necessary for this publication have been omitted (e.g., exact age and detailed gene mutations). Median age 51 years (range 16–86 years). Gene names are not considered acronyms and, therefore, are not expanded here. CMT, Charcot-Marie-Tooth neuropathy; ERT, Enzyme Replacement Therapy; F, Female; GSD, Glycogen Storage Disease; HSP, Hereditary Spastic Paraparesis; M, male; MNGIE, Mitochondrial Neurogastrointestinal Encephalomyopathy; mtDNA s.d., Mitochondrial DNA single deletion; MRS, Modified Rankin Scale; NBIA, Neurodegeneration with Brain Iron Accumulation; NCL, neuronal ceroid lipofuscinosis; NIV, Non-Invasive Ventilation; PEO, Progressive External Ophthalmoplegia; PKU, Phenylketonuria; (*) Neurological follow-up for these most severely affected patients was provided at home.

Main Phenotype	Gene	Age (Decade)	Sex	MRS	Notes
Asymptomatic	*MT-ATP6*	6th	F	0	
Asymptomatic	*PAH6th*	4th	F	0	Precociously treated PKU
Asymptomatic	*MT-ND4*	3rd	F	0	
Asymptomatic	*MT-ND4*	2nd	F	0	
Asymptomatic	*MT-ND4*	8th	F	0	
Asymptomatic	*MT-RNR1*	5th	M	0	
Asymptomatic	*MT-RNR1*	5th	F	0	
Ataxia	*SPG7*	7th	M	3	
Ataxia	*MT-ATP6*	8th	F	1	
Ataxia	*COQ8A*	9th	F	5	Mild cognitive impairment (*)
Ataxia, HSP	*SPG7*	6th	F	2	
Chorea	*GLRA1*	7th	F	1	Emotional stimulus-sensitive movements
Chorea	*VPS13A*	6th	M	3	Acanthocytosis, NIV (night)
HSP	*KIF1A*	6th	F	3	
HSP	*KIF1A3rd*	3rd	F	1	
HSP	*KIF5A*	7th	M	2	
HSP	*CYP7B1*	5th	F	4	
HSP	*KIF5A*	3rd	F	1	
HSP	*KIF5A*	8th	F	2	
Lower motor neuron syndrome	*AR*	7th	M	2	Kennedy disease
MNGIE-like	*LIG3*	5th	F	4	Neuropathy, emaciation
Myopathy	*GAA*	5th	M	1	GSD2 (Pompe); under ERT
Myopathy	*GBE1*	3rd	M	2	GSD4
Myopathy	*PYGM*	8th	F	1	GSD5 (McArdle)
Myopathy	*GAA*	9th	F	4	GSD2 (Pompe); deceased after last evaluation
Myopathy	*ACADS*	3rd	F	1	
Neuropathy	*MFN2*	7th	F	2	CMT2A
Neuropathy	*MFN2*	6th	F	2	CMT2A
Neuropathy	*MFN2*	9th	F	4	CMT2A
Optic neuropathy	*MT-ND4*	5th	F	1	Leber disease
PEO	*TWNK*	6th	F	1	
PEO	*ANT1*	7th	F	1	
PEO	*ANT1*	7th	F	1	
PEO	*MtDNA s.d.*	6th	M	1	
PEO	*TWNK*	7th	M	1	
PEO	*ANT1*	4th	M	1	
PEO	*ANT1*	4th	F	1	
Severe encephalopathy	*TMEM70*	3rd	M	5	Seizures, dysphagia, NIV (night), spastic tetraplegia, cognitive impairment (*)
Severe encephalopathy	*CLN5*	3rd	M	5	NCL; Seizures, spastic tetraplegia, cognitive impairment, blindness, myoclonus, gastrostomy (*)
Severe encephalopathy	*FXN*	3rd	M	5	Friedreich’s ataxia; deceased after last evaluation
Severe encephalopathy	*PAH*	5th	F	5	PKU; cognitive impairment (*)
Severe encephalopathy	*SUMF1*	4th	M	5	Dysphagia, spastic tetraplegia, cognitive impairment (*)
Severe encephalopathy	*PANK2*	6th	F	5	NBIA; involuntary movements, gastrostomy, NIV, cognitive impairment (*)

## Data Availability

No new data were created or analyzed in this study.

## References

[1] Cuenca-Gómez J.Á., Lara-Rojas C.M., Bonilla-López A. (2024). Cardiac manifestations in inherited metabolic diseases. Curr. Probl. Cardiol..

[2] Fernández-Eulate G., Carreau C., Benoist J.F., Lamari F., Rucheton B., Shor N., Nadjar Y. (2022). Diagnostic approach in adult-onset neurometabolic diseases. J. Neurol. Neurosurg. Psychiatry.

[3] Lopriore P., Vista M., Tessa A., Giuntini M., Caldarazzo Ienco E., Mancuso M., Siciliano G., Santorelli F.M., Orsucci D. (2024). Primary Coenzyme Q10 Deficiency-Related Ataxias. J. Clin. Med..

[4] Sechi A., Urban M.L., Murphy E., Pession A., Scarpa M., SIMMESN Adult Metabolic Working Group and of MetabERN (2024). Towards needed improvements in inherited metabolic medicine in adulthood: The SIMMESN adult metabolic working group and MetabERN Joint Position Statement. Nutr. Metab. Cardiovasc. Dis..

[5] Ferreira C.R., Rahman S., Keller M., Zschocke J., ICIMD Advisory Group (2021). An international classification of inherited metabolic disorders (ICIMD). J. Inherit. Metab. Dis..

[6] Orsucci D., Caldarazzo Ienco E., Lopriore P., Mancuso M. (2025). Disease registries and rare disorders: The virtuous example of mitochondrial medicine. Exp. Neurol..

[7] Pizzamiglio C., Mahroo O.A., Khan K.N., Patasin M., Quinlivan R. (2021). Phenotype and genotype of 197 British patients with McArdle disease: An observational single-centre study. J. Inherit. Metab. Dis..

[8] Angelini C., Burlina A., Blau N., Ferreira C.R. (2022). Clinical and biochemical footprints of inherited metabolic disorders: X. Metabolic myopathies. Mol. Genet. Metab..

[9] Orsucci D., Caldarazzo Ienco E., Rossi A., Siciliano G., Mancuso M. (2021). Mitochondrial Syndromes Revisited. J. Clin. Med..

[10] Savvatis K., Vissing C.R., Klouvi L., Florian A., Rahman M., Béhin A., Fayssoil A., Masingue M., Stojkovic T., Bécane H.M. (2022). Cardiac Outcomes in Adults With Mitochondrial Diseases. J. Am. Coll. Cardiol..

[11] Abati E., Rizzuti M., Anastasia A., Comi G.P., Corti S., Rizzo F. (2024). Charcot-Marie-Tooth type 2A in vivo models: Current updates. J. Cell. Mol. Med..

[12] Hornemann T., Penno A., Richard S., Nicholson G., van Dijk F.S., Rotthier A., Timmerman V., von Eckardstein A. (2009). A systematic comparison of all mutations in hereditary sensory neuropathy type I (HSAN I) reveals that the G387A mutation is not disease associated. Neurogenetics.

[13] Cotrina M.L., Morgenstein B., Perez M., Norcliffe-Kaufmann L., Palma J.A., Kaufmann H. (2023). Height, weight, and body mass index in patients with familial dysautonomia. PLoS ONE.

[14] Filosto M., Cotti Piccinelli S., Caria F., Gallo Cassarino S., Baldelli E., Galvagni A., Volonghi I., Scarpelli M., Padovani A. (2018). Mitochondrial Neurogastrointestinal Encephalomyopathy (MNGIE-MTDPS1). J. Clin. Med..

[15] Bonora E., Chakrabarty S., Kellaris G., Tsutsumi M., Bianco F., Bergamini C., Ullah F., Isidori F., Liparulo I., Diquigiovanni C. (2021). Biallelic variants in *LIG3* cause a novel mitochondrial neurogastrointestinal encephalomyopathy. Brain.

[16] Manzano R., Sorarú G., Grunseich C., Fratta P., Zuccaro E., Pennuto M., Rinaldi C. (2018). Beyond motor neurons: Expanding the clinical spectrum in Kennedy’s disease. J. Neurol. Neurosurg. Psychiatry.

[17] Garanto A., Ferreira C.R., Boon C.J.F., van Karnebeek C.D.M., Blau N. (2022). Clinical and biochemical footprints of inherited metabolic disorders. VII. Ocular phenotypes. Mol. Genet. Metab..

[18] Kirkman M.A., Yu-Wai-Man P., Korsten A., Leonhardt M., Dimitriadis K., De Coo I.F., Klopstock T., Chinnery P.F. (2009). Gene–environment interactions in Leber hereditary optic neuropathy. Brain.

[19] Lopez Sanchez M.I.G., Kearns L.S., Staffieri S.E., Clarke L., McGuinness M.B., Meteoukki W., Samuel S., Ruddle J.B., Chen C., Fraser C.L. (2021). Establishing risk of vision loss in Leber hereditary optic neuropathy. Am. J. Hum. Genet..

[20] Yu-Wai-Man P., Griffiths P.G., Gorman G.S., Lourenco C.M., Wright A.F., Auer-Grumbach M., Toscano A., Musumeci O., Valentino M.L., Caporali L. (2010). Multi-system neurological disease is common in patients with *OPA1* mutations. Brain.

[21] Bakhos D., Blasco H., Galvin J.J., Ferreira C.R., Blau N. (2022). Clinical and biochemical footprints of inherited metabolic diseases. IX. Metabolic ear disease. Mol. Genet. Metab..

[22] Yelverton J.C., Arnos K., Xia X.J., Nance W.E., Pandya A., Dodson K.M. (2013). The clinical and audiologic features of hearing loss due to mitochondrial mutations. Otolaryngol. Head Neck Surg..

[23] Zhan F., Zhang C., Wang S., Zhu Z., Chen G., Zhao M., Cao L. (2020). Excessive Startle with Novel *GLRA1* Mutations in 4 Chinese Patients and a Literature Review of *GLRA1*-Related Hyperekplexia. J. Clin. Neurol..

[24] Giuntini M., Picchi L., Cafforio G., Trovato R., Santorelli F.M., Vista M., Orsucci D. (2025). A Novel Variant in *GLRA1* Associated With Emotional Stimulus-Sensitive Hemichoreic Movements. Am. J. Med. Genet. A.

[25] Peacock D.J.S.J., Ferreira C.R., Horvath G., Hoffmann G.F., Blau N., Ebrahimi-Fakhari D. (2025). Clinical and biochemical footprints of inherited metabolic diseases: Ia. Movement disorders, updated. Mol. Genet. Metab..

[26] Vaisfeld A., Bruno G., Petracca M., Bentivoglio A.R., Servidei S., Vita M.G., Bove F., Straccia G., Dato C., Di Iorio G. (2021). Neuroacanthocytosis Syndromes in an Italian Cohort: Clinical Spectrum, High Genetic Variability and Muscle Involvement. Genes.

[27] Montano V., Orsucci D., Carelli V., La Morgia C., Valentino M.L., Lamperti C., Marchet S., Musumeci O., Toscano A., Primiano G. (2021). Adult-onset mitochondrial movement disorders: A national picture from the Italian Network. J. Neurol..

[28] Eshaghi K., Rao P.H., Shen M.M., Lynch D.R. (2025). Genetic and Phenotypic Variability in Siblings With Friedreich Ataxia. Neurol. Genet..

[29] Muthusamy K., Sivadasan A., Dixon L., Sudhakar S., Thomas M., Danda S., Wszolek Z.K., Wierenga K., Dhamija R., Gavrilova R. (2023). Adult-onset leukodystrophies: A practical guide, recent treatment updates, and future directions. Front. Neurol..

[30] Romani I., Sarti C., Nencini P., Pracucci G., Zedde M., Cianci V., Nucera A., Moller J., Orsucci D., Toni D. (2024). Prevalence of Fabry disease and GLA variants in young patients with acute stroke: The challenge to widen the screening. The Fabry-Stroke Italian Registry. J. Neurol. Sci..

[31] Ng Y.S., Bindoff L.A., Gorman G.S., Klopstock T., Kornblum C., Mancuso M., McFarland R., Sue C.M., Suomalainen A., Taylor R.W. (2021). Mitochondrial disease in adults: Recent advances and future promise. Lancet Neurol..

[32] Latzer I.T., Blau N., Ferreira C.R., Pearl P.L. (2023). Clinical and biochemical footprints of inherited metabolic diseases. XV. Epilepsies. Mol. Genet. Metab..

[33] Ticci C., Sicca F., Ardissone A., Bertini E., Carelli V., Diodato D., Di Vito L., Filosto M., La Morgia C., Lamperti C. (2020). Mitochondrial epilepsy: A cross-sectional nationwide Italian survey. Neurogenetics.

[34] Latzer I.T., Bertoldi M., Blau N., DiBacco M.L., Elsea S.H., García-Cazorla À., Gibson K.M., Gropman A.L., Hanson E., Hoffman C. (2024). Consensus guidelines for the diagnosis and management of succinic semialdehyde dehydrogenase deficiency. Mol. Genet. Metab..

[35] Zhang Y., Du B., Zou M., Peng B., Rao Y. (2025). Neuronal Ceroid Lipofuscinosis—Concepts, Classification, and Avenues for Therapy. CNS Neurosci. Ther..

[36] Willemsen M.A., Harting I., Wevers R.A. (2016). Neurometabolic disorders: Five new things. Neurol. Clin. Pract..

[37] van Swieten J.C., Koudstaal P.J., Visser M.C., Schouten H.J., van Gijn J. (1988). Interobserver agreement for the assessment of handicap in stroke patients. Stroke.

